# Screening and Identification of Brain Pericyte‐Selective Markers

**DOI:** 10.1111/cns.70247

**Published:** 2025-02-06

**Authors:** Minkyung Kang, Ava Nasrollahi, Feng Cheng, Yao Yao

**Affiliations:** ^1^ Department of Molecular Pharmacology and Physiology, Morsani College of Medicine University of South Florida Tampa Florida USA; ^2^ Department of Pharmaceutical Science, College of Pharmacy University of South Florida Tampa Florida USA

**Keywords:** genetic tool, PDGFRβ, pericytes, SM22α, vascular smooth muscle cells

## Abstract

**Background:**

Pericytes, a type of mural cells, exert important functions in the CNS. One major challenge in pericyte research is the lack of pericyte‐specific and subpopulation‐specific markers.

**Methods:**

To address this knowledge gap, we first generated a novel transgenic mouse line in which vascular smooth muscle cells (vSMCs) are permanently labeled with tdTomato. Next, we isolated PDGFRβ^+^tdTomato^−^ pericytes and PDGFRβ^+^tdTomato^+^ vSMCs from the brains of these mice and subsequently performed RNAseq analysis to identify pericyte‐enriched genes.

**Results:**

Using this approach, we successfully identified 40 pericyte‐enriched genes and 158 vSMC‐enriched genes, which are involved in different biological processes and molecular functions. Using ISH/IHC analysis, we found that *Pla1a* and *Cox4i2* were predominantly enriched in subpopulations of brain pericytes, although they also marked some non‐vascular parenchymal cells.

**Conclusions:**

These findings suggest that *Pla1a* and *Cox4i2* preferably label subpopulations of pericytes in the brain compared to vSMCs, and thus, they may be useful in distinguishing these populations.

## Graphical abstract

Using an innovative genetic tool coupled with FACS and RNAseq, we identified *Pla1a* and *Cox4i2* as two genes selectively enriched in brain pericytes compared to vascular smooth muscle cells.

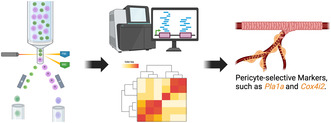

**Abbreviations**
FACS: fluorescence‐activated cell sorting
FC: fold change
FPKM: fragments per kilobase of transcript per million mapped reads
IHC: immunohistochemistry
ISH: in situ hybridization
PCA: principal component analysis
vSMCs: vascular smooth muscle cells

## 1 Introduction

The vasculature is covered by mural cells, which include pericytes and vascular smooth muscle cells (vSMCs). Pericytes are found in small blood vessels, while vSMCs predominantly reside in large blood vessels [1]. Interestingly, a higher pericyte density is found in the CNS [1]. Echoing this finding, pericytes play an indispensable role in blood–brain barrier maintenance [1, 2, 3]. In addition, pericytes are also involved in blood flow regulation, angiogenesis, and disease modulation [1, 4, 5, 6, 7, 8, 9]. Consistent with these important roles, pericyte defects have been reported in various neurological diseases, including stroke and neurodegenerative disorders. For example, altered pericyte biology (proliferation, survival, and migration) is observed in stroke brains [4, 10, 11, 12, 13, 14, 15, 16, 17]. Reduced pericyte number is observed in both rodents and humans with Alzheimer's disease [18, 19, 20, 21]. A thorough understanding of pericyte biology and function will provide insights into disease pathogenesis.

One challenge in pericyte research is the lack of pericyte‐specific markers. Although pericytes and vSMCs cover different segments of the vascular tree, they share many cellular markers [1]. For example, many widely used pericyte markers, including PDGFRβ and CD13, are not pericyte specific—they label both pericytes and vSMCs [1, 22]. Thus, additional criteria, such as vessel size (< 8 μm), have to be used to identify pericytes in vivo. In addition, most pericyte cultures likely contain vSMCs and possibly other contaminating cells. This is a major challenge in the field. To address this issue, we generated a genetic tool that enabled the isolation of pericytes and vSMCs from brain tissue, performed RNAseq analysis, and screened for pericyte‐selective markers.

## 2 Materials and Methods

### 2.1 Animals

To genetically label vSMCs with tdTomato, we crossed the SM22α‐Cre line (JAX: 004746) with the Ai14 reporter mice (JAX: 007914), which contain a floxed STOP sequence before the reporter gene tdTomato. The resulting Ai14^+/−^:SM22α‐Cre^+^ mice (both genders) at the age of 3–4 months were used for experiments. The mice were maintained in the vivarium of the University of South Florida with free access to water and food under a 12 h/12 h light/dark cycle. All procedures were approved by the Institutional Animal Care and Use Committee in accordance with the National Institutes of Health Guidelines for the Care and Use of Laboratory Animals.

### 2.2 Single‐Cell Suspension Preparation

Mouse brains were prepared using a well‐established protocol [17, 23]. Briefly, animals were anesthetized and transcardially perfused with PBS. The brains were dissected out and minced using sterile blades. The minced brain samples were then digested in 2 mg/mL Collagenase/Dispase (Roche, 11097113001), 0.5 mg/mL Elastase, and 25 μg/mL DNase I (Sigma, D5319) at 37°C for 40 min with rotation. Next, the tissue suspension was triturated with 1‐mL pipette tips, passed through a 100‐μm cell strainer (Fisher Scientific), and centrifuged at 1500 rpm for 10 min. The pellet was resuspended in 22% Percoll (GE Healthcare, 17‐0891‐02) and centrifuged at 2000 rpm for 15 min to remove myelin/debris. Red blood cells (RBCs) were removed by resuspending the pellet in RBC lysis buffer followed by centrifugation at 1500 rpm for 6 min. The pellet was resuspended in sorting buffer (HBSS + 2% FBS + 2 mM EDTA + 1% PS).

### 2.3 Fluorescence‐Activated Cell Sorting (FACS)

The single cells were incubated with PDGFRβ‐APC (eBioscience, 17‐1402‐82), CD31‐FITC (Biolegend, 103108), and CD45‐FITC (eBioscience, 11‐0451‐85) in sorting buffer for 30 min on ice. After washing in ice‐cold sorting buffer, the cells were resuspended in sorting buffer containing DAPI and filtered through a 40‐μm cell strainer. Next, samples were run through a MoFlo Astrios EQ (Beckman Coulter) cell sorter. Compensation was performed by using single color controls, and gating boundaries were set by using FMO controls. Pericytes were gated as DAPI^low^FITC^−^APC^+^tdTomato^−^ cells, and vSMCs were gated as DAPI^low^FITC^−^APC^+^tdTomato^+^ cells.

### 2.4 RNAseq and Data Analyses

Total RNA was extracted using the TRIzol reagent (Invitrogen, 15596018) and RNeasy Plus Mini Kit (Qiagen, 74136). Samples that passed RNA quality control were subjected to Ultra‐Low Input RNAseq at GENWIZ. Briefly, reverse transcription and cDNA amplification were performed using the SMART‐Seq v4 Ultra Low Input RNA Kit (Clontech), followed by library construction using the Illumina Nextera XT kit. Sequencing was performed on the Illumina platform with 2 × 150 bp paired‐end configuration. Raw RNAseq data were provided in FASTQ format. The quality of raw RNA‐seq reads was assessed using the FastQC program [24]. High‐quality reads were aligned to the mouse reference genome (NCBI Build 37) using Bowtie2 [25] and Tophat [26] with default parameters and the Ensembl Gene transfer format (GTF) file. The Cufflinks program [27, 28] was used to estimate gene expression levels in terms of FPKM (fragments per kilobase of transcript per million mapped reads). For differential expression analysis, the Cuffdiff program [27, 28] was employed to compare gene expression levels between pericytes and vSMCs, identifying differentially expressed genes. Cuffdiff does not directly assume a normal distribution for gene expression data. Instead, it models expression variability using the beta‐negative binomial distribution to estimate variance in gene expression levels and identify significant differences between conditions. These genes were input into the Shinygo web service [28, 29] for functional or pathway enrichment analysis. The principal component analysis (PCA) plot was generated using the CummeRbund [28] program. The volcano plot was created using the Tmisc package in R programming language.

### 2.5 Immunohistochemistry (IHC)

IHC was performed as described previously. Briefly, brain sections were fixed in 4% PFA for 20 min, followed by washing in PBS and incubation in blocking buffer (5% normal donkey serum in PBS + 1% BSA + 0.3% Triton X‐100) for 2 h at room temperature. Next, the sections were incubated in primary antibodies overnight at 4°C, followed by appropriate secondary antibodies for 2 h at room temperature before mounted on Fluoromount‐G with DAPI. The following primary antibodies were used: rabbit anti‐PDGFRβ (Cell Signaling Technology: 3169S), rat anti‐PDGFRβ (eBioscience: 14‐1402‐82), rat anti‐CD13 (Bio‐Rad: MCA2183), mouse anti‐smooth muscle actin‐α (SMA, MilliporeSigma: F3777), rabbit anti‐SM22α (transgelin, Genetex: GTX113561), rabbit anti‐NeuN (Abcam: AB177487), rabbit anti‐GFAP (Invitrogen: PA1‐10019), rabbit anti‐Iba‐1 (Wako: 019–19,741), and mouse anti‐Olig2 (Millipore: MABN50).

### 2.6 In Situ Hybridization (ISH)/IHC

ISH/IHC co‐staining was performed as described previously [30]. Briefly, ISH was performed on 20‐μm thick sections using the RNAscope multiplex fluorescent reagent kit V2 (Advanced Cell Diagnostics, 323100), following the manufacturer's instructions. The specific probes used in this study are: *Pla1a* (Cat #: 468021) and *Cox4i2* (Cat #: 497901‐C3). Next, the slides were washed sequentially in RNAscope Wash Buffer, PBS, and 0.5% PBST, followed by the standard IHC procedure as described above.

### 2.7 Image Analyses

Images were taken using a Nikon Eclipse Ti microscope or an LSM710 confocal microscope. Image processing was performed using ImageJ (NIH) and Adobe Photoshop.

## 3 Results

### 3.1 Isolation of Pericytes and vSMCs From Mouse Brains

**FIGURE 1:**
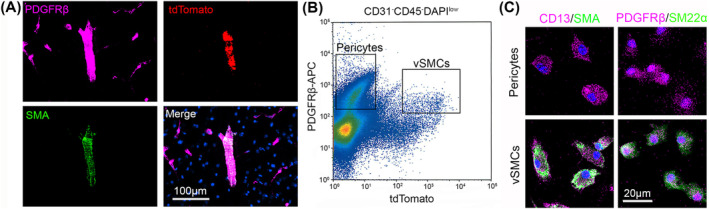
Visualization and isolation of brain pericytes and vSMCs from Ai14^+/−^;SM22α‐Cre^+^ mice. (A) tdTomato and SMA co‐localized with PDGFRβ in large blood vessels but not capillaries in mouse brains. (B) Representative flow cytometry plot showing pericytes (PDGFRβ^+^tdTomato^−^) and vSMCs (PDGFRβ^+^tdTomato^+^). (C) Isolated PDGFRβ^+^tdTomato^−^ pericytes expressed CD13 and PDGFRβ (mural cell markers) but were negative for SMA and SM22α (vSMC markers), while PDGFRβ^+^tdTomato^+^ vSMCs expressed both mural cell (CD13 and PDGFRβ) and vSMC (SMA and SM22α) markers.

To permanently label vSMCs with tdTomato, we generated Ai14^+/−^;SM22α‐Cre^+^ mice. Consistent with previous reports [31, 32], tdTomato expression was exclusively detected in SMA^+^ vSMCs in the brain (Figure 1a). In addition, tdTomato and SMA co‐localized with the mural cell marker PDGFRβ in large blood vessels but not in capillaries (Figure 1a). These results suggest that PDGFRβ and tdTomato can be used to distinguish vSMCs (PDGFRβ^+^tdTomato^+^ cells) and pericytes (PDGFRβ^+^tdTomato^−^ cells).

To determine if we can isolate pericytes and vSMCs from mouse brains by flow cytometry, we performed FACS using single‐cell suspension prepared from brain tissues of Ai14^+/−^;SM22α‐Cre^+^ mice at the age of 3–4 months. We first gated CD31^−^CD45^−^DAPI^low^ cells to exclude endothelial cells, hematopoietic cells, and dead cells. Pericytes and vSMCs were then gated based on PDGFRβ and tdTomato expression. A PDGFRβ^+^tdTomato^−^ population representing pericytes and a PDGFRβ^+^tdTomato^+^ population representing vSMCs were clearly separated in the flow cytometry plot (Figure 1b). FACS sorted PDGFRβ^+^tdTomato^−^ cells expressed CD13 and PDGFRβ (mural cell markers) but were negative for SMA and SM22α (vSMC markers), while FACS sorted PDGFRβ^+^tdTomato^+^ cells expressed both mural cell markers and vSMC markers (Figure 1c). These results strongly suggest that pericytes and vSMCs can be reliably and efficiently separated/isolated using this approach.

### 3.2 RNAseq Analysis of Brain Pericytes and vSMCs

**FIGURE 2:**
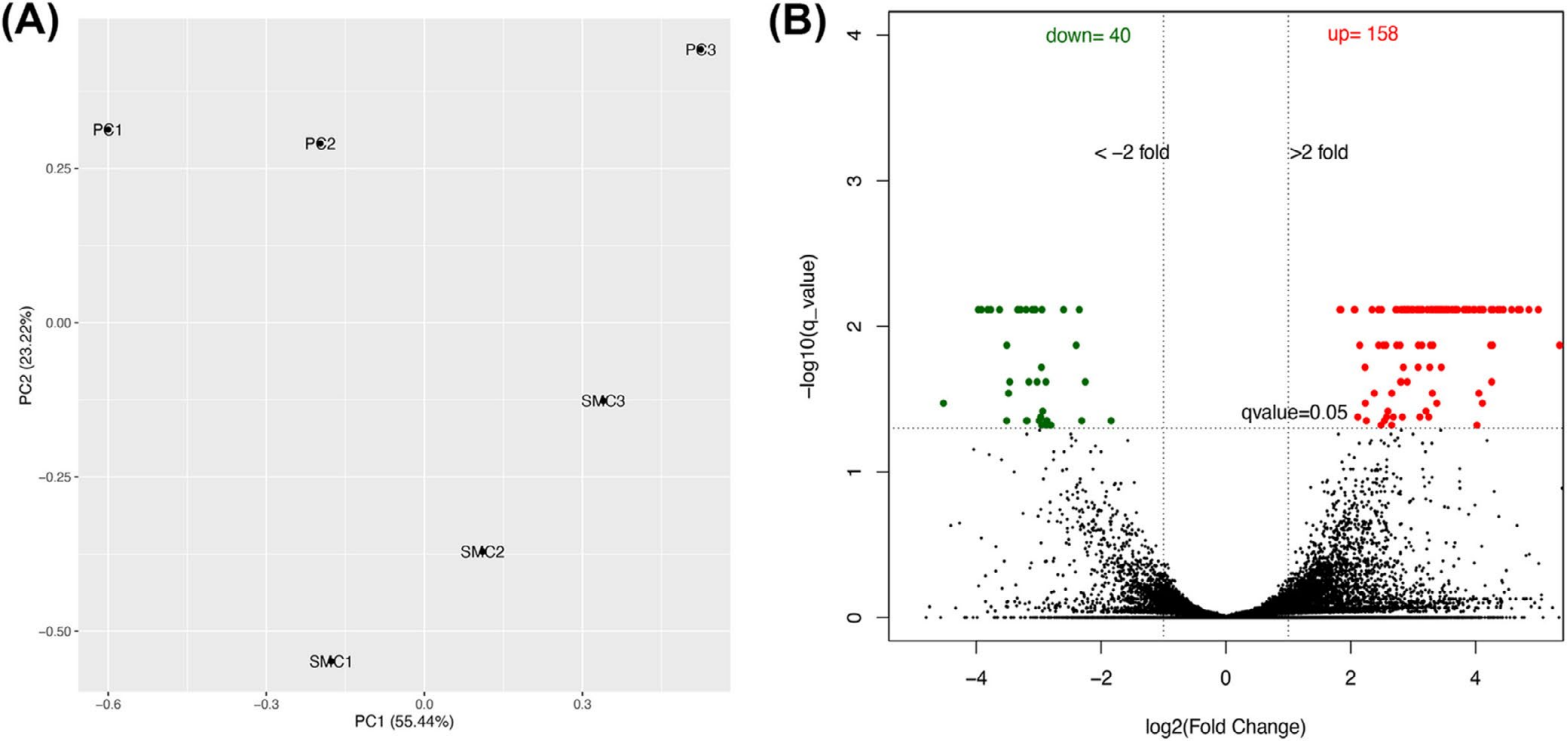
RNAseq analysis of freshly isolated brain pericytes and vSMCs. (A) Principal component analysis (PCA) demonstrated a clear separation between pericyte and vSMC samples, with some variations observed among biological replicates. (B) A comparison of the transcriptomes revealed 40 pericyte‐enriched genes and 158 vSMC‐enriched genes.

To screen for brain pericyte‐selective markers, we performed bulk RNAseq analysis using pericytes and vSMCs freshly isolated from Ai14^+/−^;SM22α‐Cre^+^ mouse brains. The principal component analysis (PCA) of all genes revealed a clear separation of pericyte samples from vSMC samples, although some variations were detected among biological replicates (Figure 2a). These data suggest that pericytes and vSMCs display distinct transcriptional profiles.

**FIGURE 3:**
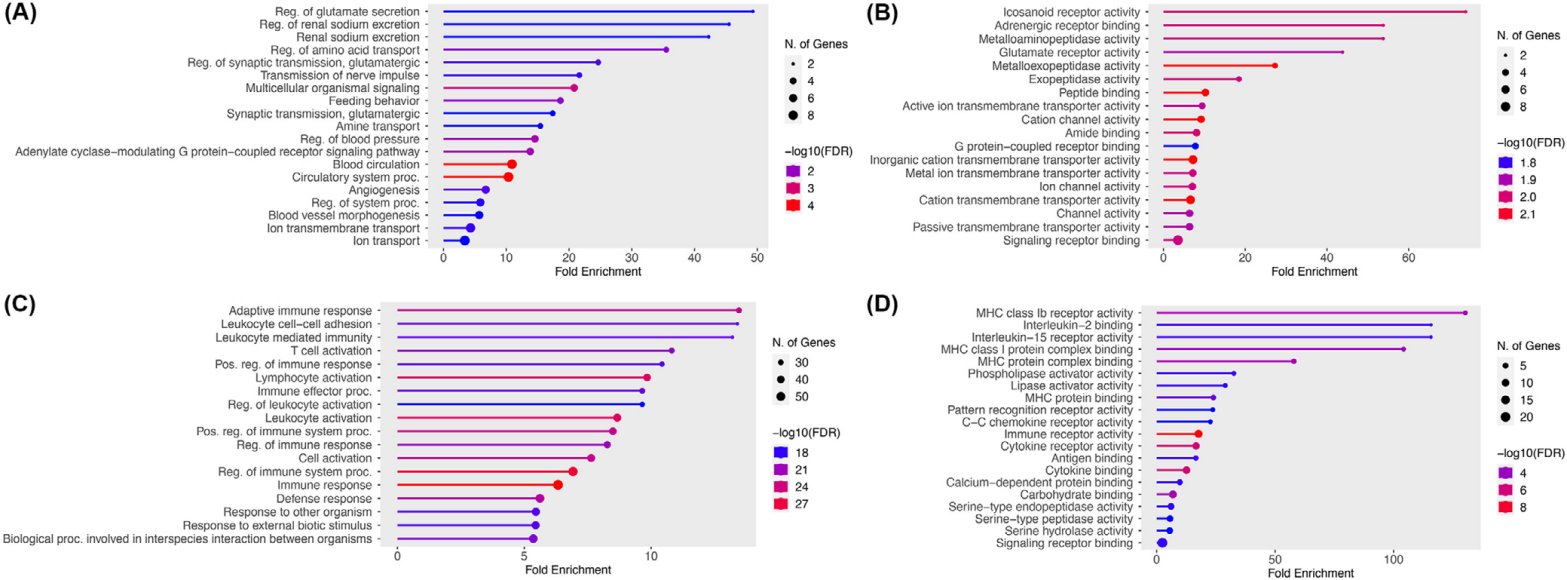
Pericyte‐enriched and vSMC‐enriched genes are involved in distinct biological processes and molecular functions. (A and B) Gene ontology analysis showing major biological processes (A) and molecular functions (B) in pericytes. (C and D) Gene ontology analysis showing major biological processes (C) and molecular functions (D) in vSMCs.

Next, we compared the transcriptomes of pericytes with that of vSMCs. Using fold change (FC) > 2 and *q* value < 0.05, we identified 40 pericyte‐enriched genes and 158 vSMC‐enriched genes (Figure 2b). Subsequent gene ontology analysis revealed that pericyte‐enriched and vSMC‐enriched genes are involved in different biological processes and molecular functions (Figure 3). Specifically, metabolic processes and receptor/peptidase functions, including glutamate secretion, renal sodium excretion, amino acid transport, multicellular organismal signaling, icosanoid receptor activity, adrenergic receptor binding, glutamate receptor activity, metalloaminopeptidase activity, metalloexopeptidase activity, and exopeptidase activity, were predominantly found in pericytes (Figure 3a,b). Interestingly, immune responses and activation, including adaptive immune response, leukocyte activation/immunity, lymphocyte activation, immune effector process, IL‐2 binding, IL‐15 receptor activity, MHC class Ib receptor activity, MHC class I protein complex binding, and MHC protein complex binding, were enriched in vSMCs (Figure 3c,d). These results strongly suggest that pericytes and vSMCs have different biological/molecular functions, justifying the need for pericyte‐ and vSMC‐selective markers.

### 3.3 Identification of Brain Pericyte‐Enriched Genes

Since various vSMC‐specific markers (SMA and Myh11) are available [31, 32, 33], we focused on pericyte‐enriched genes in this study. Among the 40 pericyte‐enriched genes (Table S1), 20 showed average transcript values of more than 25 in pericytes (Table 1). This pericyte‐enriched gene list contains several genes that were reported to be pericyte‐selective in previous studies, including *Vtn* [34, 35], *Enpep* [35, 36], *Kcnj8* [35, 36, 37], and *Abcc9* [35, 36, 37], again indicating successful separation of pericytes and vSMCs in this study.

**TABLE 1:** Pericyte‐enriched genes with average transcript values of more than 25.0 in pericytes.

Gene ID	Gene name	PC value	vSMC value	logFC	*p*	*q*
ENSMUSG00000002847	*Pla1a*	37.04	2.37	−3.97	5.00E‐05	7.68E‐03
ENSMUSG00000009876	*Cox4i2*	81.85	10.05	−3.03	2.00E‐04	2.41E‐02
ENSMUSG00000015405	*Ace2*	47.20	4.28	−3.46	2.00E‐04	2.41E‐02
ENSMUSG00000017344	*Vtn*	1586.57	142.00	−3.48	2.50E‐04	2.88E‐02
ENSMUSG00000017390	*Aldoc*	88.69	14.62	−2.60	5.00E‐05	7.68E‐03
ENSMUSG00000017417	*Plxdc1*	34.64	9.68	−1.84	4.50E‐04	4.45E‐02
ENSMUSG00000020178	*Adora2a*	26.31	3.40	−2.95	5.00E‐04	4.77E‐02
ENSMUSG00000020486	*Sept4*	366.36	73.87	−2.31	4.50E‐04	4.45E‐02
ENSMUSG00000020695	*Mrc2*	25.70	5.05	−2.35	5.00E‐05	7.68E‐03
ENSMUSG00000026424	*Gpr37l1*	56.54	6.15	−3.20	5.00E‐05	7.68E‐03
ENSMUSG00000028024	*Enpep*	42.60	5.84	−2.87	4.50E‐04	4.45E‐02
ENSMUSG00000029223	*Uchl1*	42.71	4.37	−3.29	5.00E‐05	7.68E‐03
ENSMUSG00000030247	*Kcnj8*	183.84	14.89	−3.63	5.00E‐05	7.68E‐03
ENSMUSG00000030249	*Abcc9*	56.72	4.03	−3.82	5.00E‐05	7.68E‐03
ENSMUSG00000034881	*Tbxa2r*	60.08	6.56	−3.19	4.50E‐04	4.45E‐02
ENSMUSG00000037031	*Tspan15*	26.44	5.55	−2.25	2.00E‐04	2.41E‐02
ENSMUSG00000037892	*Pcdh18*	43.53	5.90	−2.88	2.00E‐04	2.41E‐02
ENSMUSG00000056481	*Cd248*	115.34	8.47	−3.77	5.00E‐05	7.68E‐03
ENSMUSG00000072941	*Sod3*	170.00	20.46	−3.05	5.00E‐05	7.68E‐03
ENSMUSG00000076577	*Igkv8‐30*	63.87	7.68	−3.06	5.00E‐05	7.68E‐03

**FIGURE 4:**
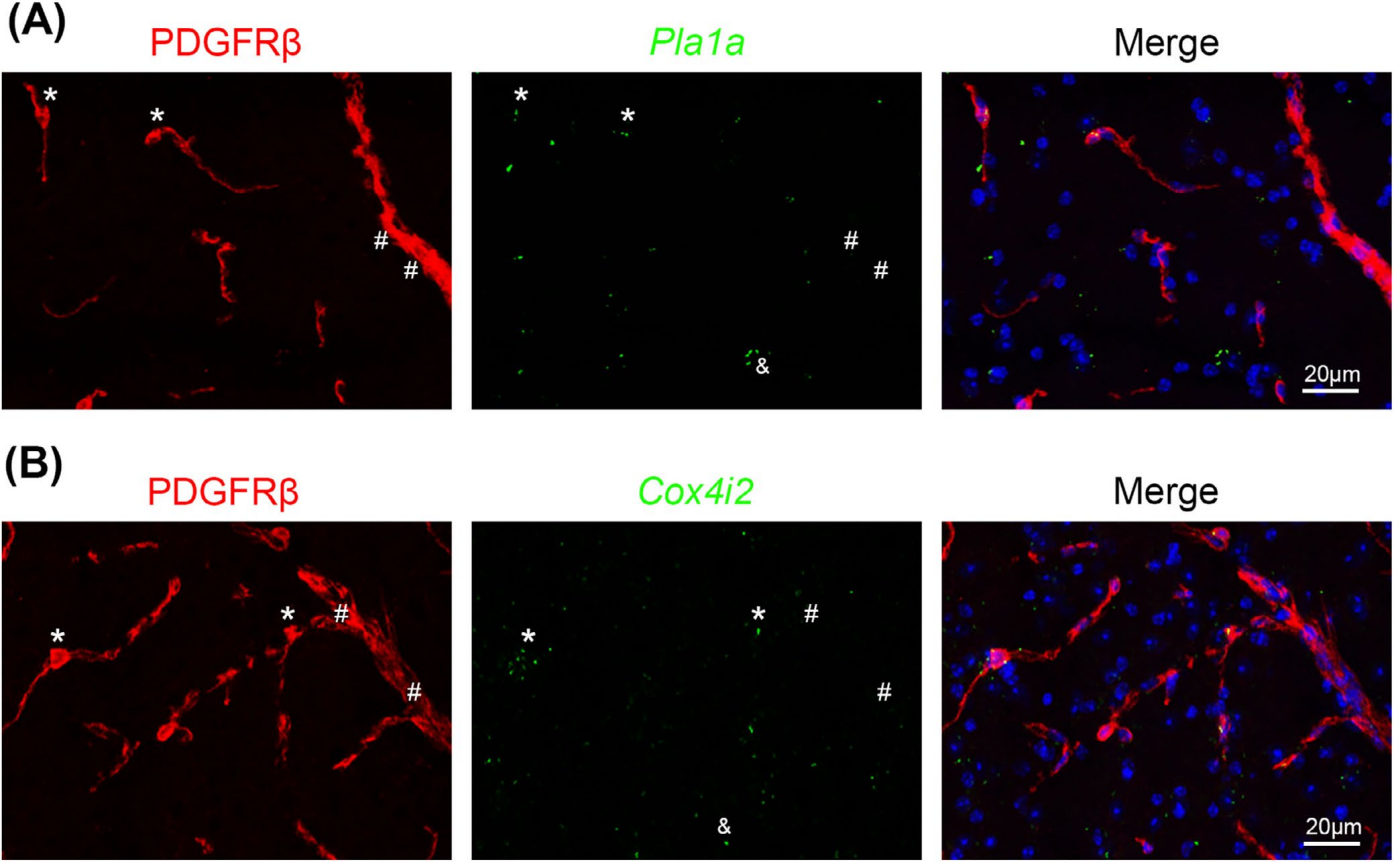
Validation of pericyte‐enriched genes in vivo. (A) *Pla1a* mRNA was detected in PDGFRβ^+^ pericytes (*) in capillaries but not in PDGFRβ^+^ vSMCs (#) in large vessels in mouse brains. (B) *Cox4i2* mRNA was predominantly found in PDGFRβ^+^ pericytes (*) in capillaries but not PDGFRβ^+^ vSMCs (#) in large vessels. Notably, *Pla1a* and *Cox4i2* failed to label all pericytes and were also present in non‐vascular parenchymal cells (&) in mouse brains.

**FIGURE 5:**
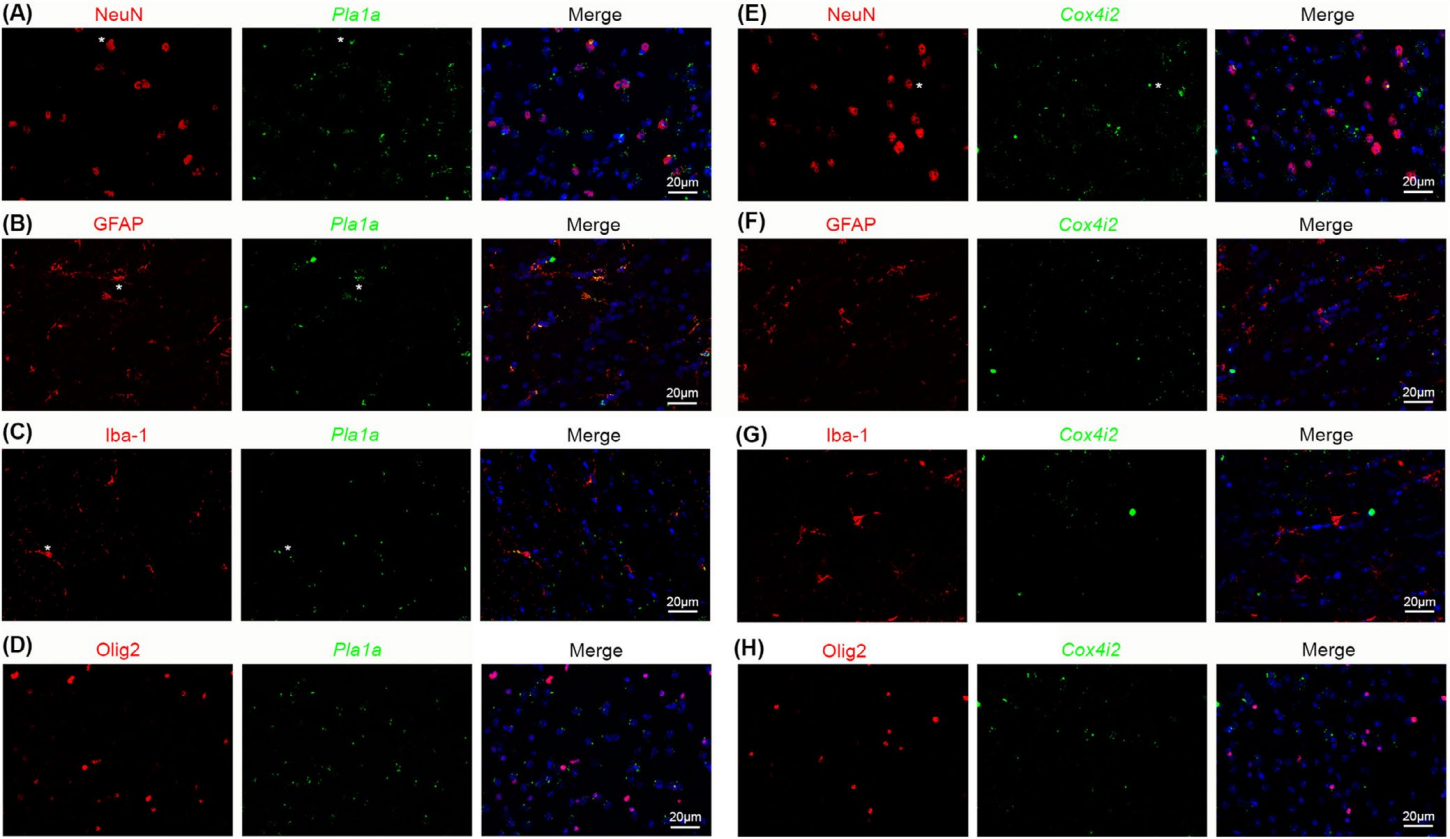
*Pla1a* and *Cox4i2* expression in non‐vascular cells in the brain. (A‐D) *Pla1a* mRNA was detected in some NeuN^+^ neurons (* in A), GFAP^+^ astrocytes (* in B), and Iba‐1^+^ microglia (* in C), but not Olig2^+^ oligodendrocytes (D) in mouse brains. (E–H) *Cox4i2* mRNA was found in some NeuN^+^ neurons (* in E), but not GFAP^+^ astrocytes (F), Iba‐1^+^ microglia (G), or Olig2^+^ oligodendrocytes (H) in mouse brains.

We further chose *Pla1a* and *Cox4i2*, two previously unidentified pericyte‐enriched genes, to validate. Specifically, we examined the expression of *Pla1a* and *Cox4i2* in PDGFRβ^+^ mural cells in mouse brain by ISH/IHC analysis. Pericytes and vSMCs were determined based on the size of blood vessels they cover: PDGFRβ^+^ cells in small (< 8 μm) and large (≥ 8 μm) blood vessels were defined as pericytes and vSMCs, respectively. Consistent with our RNAseq data, *Pla1a* mRNA was detected in PDGFRβ^+^ pericytes (*) in capillaries but not in PDGFRβ^+^ vSMCs (#) in large vessels (Figure 4a). Similarly, *Cox4i2* mRNA was predominantly found in PDGFRβ^+^ pericytes (*) in capillaries but not PDGFRβ^+^ vSMCs (#) in large vessels (Figure 4b). It should be noted, however, that *Pla1a* or *Cox4i2* did not label all pericytes in mouse brains (Figure 4), suggesting that these markers may label subpopulations of pericytes. In addition to blood vessels, *Pla1a* ^+^ or *Cox4i2* ^+^ cells were also found in nonvascular cells in brain parenchyma (& in Figure 4), indicating that these markers are not pericyte‐specific. Subsequent analysis showed that *Pla1a* was detected in some neurons, astrocytes, and microglia, but not oligodendrocytes (Figure 5a–5d). Interestingly, *Cox4i2* was found in some neurons, but not astrocytes, microglia, or oligodendrocytes (Figure 5e–5h). Together, these results demonstrate that *Pla1a* and *Cox4i2* are enriched in pericytes (or their subpopulations) compared to vSMCs in mouse brains, although they are also expressed in some brain parenchymal cells. The expression of *Pla1a* and *Cox4i2* in different cells in the brain is summarized in Table 2.

**TABLE 2:** Summary of *Pla1a* and *Cox4i2* expression in mouse brain.

Genes	Expression in brain cells					
	Pericytes	vSMCs	Neurons	Astrocytes	Microglia	Oligodendrocytes
*Pla1a*	++	−	+	+	+	−
*Cox4i2*	++	−	+	−	−	−

## 4 Discussion

In this study, we isolated brain pericytes and vSMCs from Ai14^+/−^;SM22α‐Cre^+^ mice, in which vSMCs are permanently labeled with tdTomato. By comparing their transcriptional profiles, we identified brain pericyte‐selective genes. A previous single‐cell RNAseq study identified several pericyte‐selective genes, including *Kcnj8* and *Abcc9* [38]. Consistent with this report, these pericyte‐enriched genes were also found in our study, indicating the success and efficacy of our approach. In addition, we found brain pericyte‐enriched genes not reported in previous studies. Specifically, our non‐biased RNAseq and in situ hybridization analyses showed that *Pla1a* and *Cox4i2* were highly enriched in brain pericytes in capillaries compared to brain vSMCs in large blood vessels. These new brain pericyte‐selective markers may be useful in the identification and/or differentiation of pericytes and vSMCs in the brain. In addition, our multifaceted RNAseq analyses also revealed different biological processes and molecular functions in brain pericytes and vSMCs, which support and provide insights into the functional differences between these cells.

It is worth noting that some but not all pericytes are labeled by these markers. This may be due to pericyte heterogeneity [1, 9, 38]: it is possible that these markers only label subpopulations of pericytes. The specific subpopulations of pericytes marked by these markers remain unclear and need further investigation. In addition to vascular cells, *Pla1a* is also detected in some neurons and glial cells (astrocytes and microglia), while *Cox4i2* is also found in some neurons. Neither markers were detected in oligodendrocytes. The expression of *Pla1a* and *Cox4i2* in different cells in the brain is summarized in Table 2. This expression pattern is consistent with previous RNAseq studies [39, 40, 41, 42, 43]. Although not brain pericyte‐specific, these markers may still be useful in distinguishing pericytes and vSMCs when contaminating cells are absent. For example, these markers can be used to identify pericytes and vSMCs from brain blood vessels isolated by dextran floating. Additionally, they can be used in combination with vascular, neuronal, and/or glial markers to identify pericytes and vSMCs by IHC.

One limitation of this study is that it only focuses on SMA/SM22α‐negative pericytes. In addition to SMA/SM22α‐negative pericytes that cover capillaries and post‐capillary venules, a subpopulation of SMA/SM22α‐positive pericytes at pre‐capillary arterioles has also been reported [9, 38]. However, these SMA/SM22α‐positive pericytes were gated into the vSMC pool due to their expression of tdTomato. Future efforts should be made to distinguish different subpopulations of pericytes.

## Author Contributions

Y.Y. conceived the study. M.K., A.N., F.C., and Y.Y. performed the experiments, collected the data, and analyzed the data. M.K. wrote the first draft of the manuscript. All authors edited the manuscript and approved the final version of the manuscript.

## Ethics Statement

This study was performed in line with the NIH guidelines for the care and use of animals. Approval was granted by the Institutional Animal Care and Use Committee at the University of South Florida.

## Consent

The authors have nothing to report.

## Conflicts of Interest

The authors declare no conflicts of interest.

## Supporting information


**Table S1.** Pericyte‐enriched genes.

## Data Availability

All the data supporting the findings of this study are available from the corresponding author upon reasonable request.
