# Supplementary material for: Screening and Identification of Brain Pericyte‐Selective Markers
Source: CNS Neurosci Ther. 2025 Feb 6;31(2):e70247. doi: 10.1111/cns.70247 (PMC11799917; doi:10.1111/cns.70247)
Supplement: Supplementary file 1 — Table S1. Pericyte‐enriched genes. [file CNS-31-e70247-s001.docx]

**Supplementary Table 1.** **Pericyte-enriched genes.**

| **Gene ID** | **Gene Name** | **PC Value** | **vSMC Value** | **logFC** | **p_value** | **q_value** |
| --- | --- | --- | --- | --- | --- | --- |
| ENSMUSG00000000792 | Slc5a5 | 19.15 | 2.15 | -3.16 | 2.00E-04 | 2.41E-02 |
| ENSMUSG00000002847 | Pla1a | 37.04 | 2.37 | -3.97 | 5.00E-05 | 7.68E-03 |
| ENSMUSG00000004098 | Col5a3 | 5.30 | 0.73 | -2.87 | 5.00E-04 | 4.77E-02 |
| ENSMUSG00000006522 | Itih3 | 9.59 | 1.21 | -2.99 | 4.50E-04 | 4.45E-02 |
| ENSMUSG00000009876 | Cox4i2 | 81.85 | 10.05 | -3.03 | 2.00E-04 | 2.41E-02 |
| ENSMUSG00000015405 | Ace2 | 47.20 | 4.28 | -3.46 | 2.00E-04 | 2.41E-02 |
| ENSMUSG00000017344 | Vtn | 1586.57 | 142.00 | -3.48 | 2.50E-04 | 2.88E-02 |
| ENSMUSG00000017390 | Aldoc | 88.69 | 14.62 | -2.60 | 5.00E-05 | 7.68E-03 |
| ENSMUSG00000017417 | Plxdc1 | 34.64 | 9.68 | -1.84 | 4.50E-04 | 4.45E-02 |
| ENSMUSG00000019890 | Nts | 6.33 | 0.70 | -3.18 | 4.50E-04 | 4.45E-02 |
| ENSMUSG00000020178 | Adora2a | 26.31 | 3.40 | -2.95 | 5.00E-04 | 4.77E-02 |
| ENSMUSG00000020486 | Sept4 | 366.36 | 73.87 | -2.31 | 4.50E-04 | 4.45E-02 |
| ENSMUSG00000020524 | Gria1 | 2.61 | 0.49 | -2.40 | 1.00E-04 | 1.35E-02 |
| ENSMUSG00000020695 | Mrc2 | 25.70 | 5.05 | -2.35 | 5.00E-05 | 7.68E-03 |
| ENSMUSG00000021943 | Gdf10 | 9.63 | 1.12 | -3.10 | 5.00E-05 | 7.68E-03 |
| ENSMUSG00000022658 | Tagln3 | 18.55 | 2.41 | -2.95 | 5.00E-05 | 7.68E-03 |
| ENSMUSG00000022754 | Tmem45a | 19.36 | 2.48 | -2.97 | 4.00E-04 | 4.20E-02 |
| ENSMUSG00000026424 | Gpr37l1 | 56.54 | 6.15 | -3.20 | 5.00E-05 | 7.68E-03 |
| ENSMUSG00000028024 | Enpep | 42.60 | 5.84 | -2.87 | 4.50E-04 | 4.45E-02 |
| ENSMUSG00000028031 | Dkk2 | 2.75 | 0.35 | -2.96 | 1.50E-04 | 1.91E-02 |
| ENSMUSG00000029223 | Uchl1 | 42.71 | 4.37 | -3.29 | 5.00E-05 | 7.68E-03 |
| ENSMUSG00000030247 | Kcnj8 | 183.84 | 14.89 | -3.63 | 5.00E-05 | 7.68E-03 |
| ENSMUSG00000030249 | Abcc9 | 56.72 | 4.03 | -3.82 | 5.00E-05 | 7.68E-03 |
| ENSMUSG00000032281 | Acsbg1 | 12.86 | 1.27 | -3.34 | 5.00E-05 | 7.68E-03 |
| ENSMUSG00000032492 | Pth1r | 23.90 | 2.84 | -3.07 | 5.00E-05 | 7.68E-03 |
| ENSMUSG00000033470 | Cysltr2 | 11.78 | 1.69 | -2.80 | 5.00E-04 | 4.77E-02 |
| ENSMUSG00000034881 | Tbxa2r | 60.08 | 6.56 | -3.19 | 4.50E-04 | 4.45E-02 |
| ENSMUSG00000037031 | Tspan15 | 26.44 | 5.55 | -2.25 | 2.00E-04 | 2.41E-02 |
| ENSMUSG00000037171 | Nodal | 19.37 | 1.28 | -3.92 | 5.00E-05 | 7.68E-03 |
| ENSMUSG00000037892 | Pcdh18 | 43.53 | 5.90 | -2.88 | 2.00E-04 | 2.41E-02 |
| ENSMUSG00000039062 | Anpep | 9.89 | 1.29 | -2.93 | 3.50E-04 | 3.82E-02 |
| ENSMUSG00000040373 | Cacng5 | 2.69 | 0.24 | -3.51 | 4.50E-04 | 4.45E-02 |
| ENSMUSG00000056481 | Cd248 | 115.34 | 8.47 | -3.77 | 5.00E-05 | 7.68E-03 |
| ENSMUSG00000056755 | Grm7 | 14.43 | 1.27 | -3.51 | 1.00E-04 | 1.35E-02 |
| ENSMUSG00000063297 | Luzp2 | 10.22 | 1.02 | -3.33 | 5.00E-05 | 7.68E-03 |
| ENSMUSG00000072941 | Sod3 | 170.00 | 20.46 | -3.05 | 5.00E-05 | 7.68E-03 |
| ENSMUSG00000074199 | Krtdap | 2.14 | 0.00 | -Inf | 1.50E-04 | 1.91E-02 |
| ENSMUSG00000074555 | Gm10714 | 9.99 | 0.43 | -4.53 | 3.00E-04 | 3.37E-02 |
| ENSMUSG00000076577 | Igkv8-30 | 63.87 | 7.68 | -3.06 | 5.00E-05 | 7.68E-03 |
| ENSMUSG00000087067 | Gm11532 | 0.87 | 0.12 | -2.87 | 4.50E-04 | 4.45E-02 |
